# Are Older Adults More Prosocial Than Younger Adults? A Systematic Review and Meta-Analysis

**DOI:** 10.1093/geront/gnae082

**Published:** 2024-07-04

**Authors:** Duo Li, Yuan Cao, Bryant P H Hui, David H K Shum

**Affiliations:** Department of Rehabilitation Sciences, The Hong Kong Polytechnic University, Hong Kong, China; Department of Social Work and Social Administration, The University of Hong Kong, Hong Kong, China; Department of Applied Social Sciences, The Hong Kong Polytechnic University, Hong Kong, China; Department of Rehabilitation Sciences, The Hong Kong Polytechnic University, Hong Kong, China; Research Institute for Smart Ageing, The Hong Kong Polytechnic University, Hong Kong, China

**Keywords:** Age difference, Altruism, Generosity, Prosocial behavior

## Abstract

**Background and Objectives:**

Prosociality refers to voluntary behaviors that intend to benefit others. Most of the existing literature suggests that older adults tend to act more prosocially compared to younger adults, whereas some studies show that older adults might not be that prosocial under certain conditions. The current study aimed to summarize the mixed findings and quantify the age difference in prosociality by conducting a qualitative systematic review and a quantitative meta-analysis.

**Research Design and Methods:**

Literature search was conducted based on 5 databases. The Preferred Reporting Items for Systematic Reviews and Meta-Analyses guidelines were followed and this review was registered at PROSPERO (CRD42022333373).

**Results:**

Based on the qualitative synthesis of 51 studies, older adults (*n* = 109,911) were more prosocial than younger adults (*n* = 68,501). The meta-analysis of 46 studies further supported this age effect (Hedges’ *g* = 0.31, 95% confidence interval [0.24, 0.37]), and this age effect might be moderated by the types of prosociality. We discovered a moderate age effect in sharing (Hedges’ *g* = 0.53), but a nonsignificant age effect in helping (Hedges’ *g* = 0.11), comforting (Hedges’ *g* = −0.20), or mixed prosociality (Hedges’ *g* = 0.15). Additionally, the age effect was only significant when older adults had higher socioeconomic status than younger adults.

**Discussion and Implications:**

Future research should develop more comprehensive measures of prosociality, examine more variables that influence aging and prosociality, and investigate the neural mechanism(s) of prosociality to achieve a thorough understanding of the age difference in prosociality.

Prosociality refers to dispositions, motivations, and behaviors for the benefits of others (Eisenberg & Miller, 1987). Research has consistently shown that prosociality is linked to improved mental and physical health outcomes. Several meta-analyses have revealed that individuals who engage in prosocial behaviors tend to report higher levels of happiness, life satisfaction, and well-being while experiencing lower levels of stress, anxiety, and depression (Hui, Ng, et al., 2020). Given the important and beneficial roles of prosociality, it is crucial to investigate and study different levels of prosociality in diverse populations.

## Prosociality in Older Adults

As the global population is rapidly aging, researchers have increasingly turned their attention to prosociality in older adults (Bailey et al., 2021; Bhatta et al., 2021). For example, studies found that older adults were more altruistic than younger adults based on the result of self-report scales that covered different prosocial behaviors, such as the Self-Report Altruism scale (Dury et al., 2023; Sparrow et al., 2019). Older adults also shared more money with others than younger adults in a dictator game, which indicated that they acted more prosocially (Byrne et al., 2023; Nava et al., 2023). Several theories have been proposed to explain this age difference. According to the Socioemotional Selectivity Theory (Carstensen et al., 1999), the perception of time changes with age. Although younger adults perceive time as unlimited and prioritize knowledge goals, older adults perceive time as limited and prioritize emotional goals. Thus, older adults are more likely to engage in prosocial behaviors that help to achieve their emotional goals. Recently, a Value-based Decision Framework was proposed (Mayr & Freund, 2020) to understand the increased level of prosociality in older adults. In this framework, various factors that may influence decision-making on prosocial behavior are divided into distal and proximal factors. Distal factors include resources and constraints (e.g., working memory, financial resources), motivational orientations, and cultural scripts (e.g., generative concerns, social norms), whereas proximal factors include the costs (e.g., cost of money) and benefits (e.g., reciprocity) of prosocial decision-making. For instance, as older adults have more generative concerns and care more for the future generation, they are more willing to provide support to younger adults (Villar et al., 2021). The above theories have been supported by reviews focusing on the age difference in prosociality.

## Existing Reviews and Meta-Analyses

Reviews and meta-analyses have been carried out on aging and prosociality to summarize the findings on older adults. For instance, Ebner et al. (2017) qualitatively synthesized the literature on aging and prosociality and then proposed that older adults acted more prosocially despite their declined cognitive functions. Among the included studies that reported the age range of participants, older adults were considered as individuals aged 55 years and above in two studies, 60 years and above in three studies, 65 years and above in five studies, and 70 years in one study. The younger adults were considered as individuals aged 20 years in one study, 30 years and below in six studies, 35 years and below in two studies, and 45 years and below in two studies. Their review pointed out that several factors could influence the age difference in prosociality, for example, context dependency, cultural differences, and neural activation. Similarly, Isaacowitz et al.’s (2021) review proposed an age-related prosocial shift in that older adults focus more on others’ welfare than their own. This might be explained by their prioritization of emotional goals, stronger altruistic motives, and increased generative concern. By quantifying the age difference in prosociality, a recent meta-analysis (Sparrow et al., 2021) found a large mean age effect in 16 studies that suggested older adults who aged 60 years or above were more altruistic than younger adults who aged 35 years or below (Hedges’ *g* = 0.61). Despite providing insightful findings, this meta-analysis is not without limitations. Firstly, it focused only on altruistic behaviors that were motivated solely by participants’ desire to help others, even at their own expense. However, real-life prosocial behaviors can include a broader range of acts that are motivated by reciprocity, cooperation, or reputational concerns (Pfattheicher et al., 2022). Secondly, the number of studies (*k* = 16) is relatively small. Thirdly, studies conducted in the United States and Canada were overrepresented, whereas those conducted in other countries, such as Estonia and Thailand (Mõttus et al., 2015; Nakavachara, 2018), were not included. Thus, future meta-analyses should cover more studies from other countries and delve further into diverse types of prosociality to achieve a better understanding of the age difference in prosociality.

## Moderators of Age Difference in Prosociality

### Types of Prosociality

Consistent with the aforementioned meta-analysis, many empirical studies have suggested that older adults are more prosocial than younger adults (e.g., older adults donated more money than younger adults, Bruine de Bruin & Ulqinaku, 2021). However, in a study where participants had the opportunity to donate nonmonetary resources such as time, older adults were less willing to donate time than younger adults (Best & Freund, 2021). This suggests that older adults may be more willing to engage in some but not all types of prosociality.

According to the developmental model of prosociality (Dunfield, 2014), this construct can be divided into three types (viz., sharing, helping, and comforting) based on the needs of the recipients. Sharing refers to the provision of solutions to others’ material needs. It is the most researched type of prosocial behavior, and studies on monetary sharing have consistently found that older adults give more money to others than younger adults (Chapman et al., 2023; Sparrow & Spaniol, 2018). Helping refers to assisting with others’ instrumental needs. In a study that examined helping, participants were asked to help compile a pamphlet for a charity, and older and younger adults showed a similar willingness to help (Bailey et al., 2020). Comforting, which refers to alleviating others’ emotional distress, has seldom been examined in older adults. In a large-sample survey (Sin et al., 2021), comforting was assessed with one question: “Did you provide emotional support to anyone for reasons related to COVID-19?,” and older adults were found to offer more emotional support. To summarize, previous studies have produced mixed results in different types of prosocial behavior, but no review has split prosocial behavior into different types.

### Measures of Prosociality

Various methodologies have been employed to explore the dimensions of prosociality, encompassing both self-report scales and behavioral tasks. For sharing, it is commonly measured by behavioral tasks such as the dictator game and donation (Albouy & Décaudin, 2018; Ogawa et al., 2020; Rieger & Mata, 2015). For helping, an ecologically valid task is asking participants if they could help to complete another task after they have finished some initial tasks (Hao & Liu, 2016). Comforting is usually measured by self-reported questions, for example, “Did you spend any time giving emotional support to anyone, like listening to their problems, giving advice, or comforting them?” (Chi et al., 2021). These different measures can contribute to mixed findings in the age difference in prosociality because older adults and younger adults may exhibit different response bias toward self-report scales. For instance, older adults might have difficulty in understanding the vague item wording thus providing potentially biased responses (Hill et al., 2019). Therefore, the current meta-analysis has also included the measure of prosociality (self-report vs behavior) as a potential moderator of age difference in prosociality.

### Socioeconomic Status

Research has found that older adults tend to be more generous as they have higher socioeconomic status (SES; Freund & Blanchard-Fields, 2014). A recent study provided evidence that SES moderated the age difference in empathic concern and further influenced prosocial tendencies through a moderated mediation effect in a continuous age range of participants (Li & Siu, 2021). Specifically, the positive association between age and empathy was found to be significant primarily among participants with high SES, whereas it was less pronounced for those with low SES. Based on these findings, the current meta-analysis tested SES as a potential moderator.

## The Current Review and Meta-Analysis

To address the limitations of existing literature, we aimed to conduct a systematic review and meta-analysis to comprehensively synthesize the divergent findings in the fields of aging and prosociality. Specifically, we intended to include a broader array of empirical studies so that we could qualitatively summarize the mixed findings and quantify the age difference in various types of prosocial behaviors. In the meta-analysis, Hedge’s *g* was estimated to compare prosociality between the older adults and younger adults. In addition, we planned to enhance our understanding and knowledge by identifying the moderators (i.e., types of prosociality, measures of prosociality, and SES of older adults) that may influence the relationship between aging and prosociality.

## Method

### Literature Search

The literature search was conducted using five databases: PsycINFO, PubMed, Web of Science, Embase, and Scopus. The key search terms included prosocial*, altruis*, cooperat*, genero*, donat*, helping, sharing, comforting, and “dictator game” coupled with synonyms of “older adults.” Only English peer-reviewed journals were included in the literature search because English prosociality literature is the largest, easily accessible, and widely recognized literature.

### Inclusion Criteria

#### Participants

Studies had to recruit at least one group of older adults (mean age > 60 years) and at least one group of younger adults (mean age < 35 years). Studies that recruited a wide range of participants (e.g., participants aged from 18 to 100 years) were included only if the older group and the younger group were compared for data analyses. Because the current review aimed to compare prosociality in older adults to younger adults, studies that only recruited older adults without comparison to younger adults (or vice versa) were excluded. Moreover, the current review focused on healthy aging population. Thus, studies focusing on clinical populations, such as people with dementia, were excluded.

#### Measures

Studies had to use at least one quantitative measurement to assess prosociality. Meanwhile, studies that adopted qualitative measurements, such as interviews, were excluded. Blood donation and organ donation studies were not included as they were considered extraordinary prosocial behavior, which was distinct from ordinary prosocial behavior (Smith, 2011). Besides, community-level prosocial behavior such as formal volunteering was not included because a scoping review had already summarized the findings on volunteering older adults (Serrat et al., 2020). Studies had to report age-related differences in prosociality in their results. Studies that measured prosociality but did not report any findings were excluded.

### Data Extraction

Data from the studies, including the characteristics of participants, details of methods, and results that were related to prosociality, were extracted using the Covidence software (https://www.covidence.org/). Two coders were assigned to extract the data independently. Inconsistencies in the extracted data were resolved through discussion. The sample size (*N*), mean (*M*), and standard deviation (*SD*) of the prosocial behavior of the younger and older groups were extracted. If the study did not report the mean and *SD*, then the *p* value, Cohen’s *d*, and other relevant statistical information were extracted to estimate effect sizes.

### Meta-Analytic Procedure

The Comprehensive Meta-Analysis (Borenstein, 2022) software was used to calculate the effect sizes of the studies (viz., Hedge’s *g*). The effect sizes were weighted by using inverse variance. Hedge’s *g* was chosen because it removes the potential bias raising from small sample sizes (Hedges & Olkin, 2014). It is suggested that Hedge’s *g* of 0.20, 0.50, and 0.80 to be small, medium, and large effects, respectively, and we used this rule to interpret the magnitude of the effect sizes (Cohen, 2013). Given the variation of the studies included, a random-effects model (DerSimonian and Laird Model) was used. For studies that recruited more than one group of older/younger adults, a combined mean and *SD* were calculated according to the Cochrane Handbook. If a study reported prosocial behavior under different conditions, the outcomes were averaged to calculate a combined effect size. Some studies did not report enough data for effect size calculation. Hence, we solicited their corresponding authors via email to seek additional data. Seventeen authors were contacted and 12 of them replied with sufficient data. The heterogeneity across the studies was assessed using three indicators: *Q* statistic, τ, and *I*^2^. Specifically, *Q* indicates the existence of heterogeneity, τ indicates the standard deviation of the true effect sizes, and *I*^2^ indicates if the total variation observed in the estimated true effect sizes is due to heterogeneity rather than sampling error. An *I*^2^ value over 50% suggests study characteristics should be examined to explain the heterogeneity across studies (Higgins & Thompson, 2002).

To evaluate variables that might influence the age difference in prosociality, certain moderators were extracted. These included demographic moderators such as participants’ mean age, education level, SES, and the country under study. Amongst them, education level and SES were coded for the older group in comparison with the younger group, that is, at a higher/lower/similar level than the younger group. Methodological moderators include the types and measures of prosociality. The type of prosociality was coded as helping, sharing, and comforting. It was coded as mixed if prosociality was measured using multiple items that involved at least two types of prosociality (e.g., scales include items on sharing such as donating goods and helping such as giving directions), or if it was measured with a general question that did not target any specific type of prosociality (e.g., Do you help others when you have a chance?). Meanwhile, the measure of prosociality was coded as either a behavioral or a self-report measure. Fixed-effect model was used to combine studies based on study-level covariates (e.g., types of prosociality) and random-effects model was used within each subgroup.

To examine the publication bias, classical fail-safe *N* analysis, trim-and-fill analysis, and Egger’s regression intercept (Egger et al., 1997) were used.

### Risk of Bias Assessment

The risk of bias of each included study was evaluated using the Quality Assessment Tool for Observational Cohort and Cross-Sectional Studies (National Institutes of Health [NIH], 2016). This tool includes 14 criteria that assess the study design, procedure, and results. Among them, four criteria (i.e., nos. 3, 7, 12, and 13) were deemed inapplicable as they were designed for cohort studies, leaving 10 criteria for our risk of bias assessment. The included studies were rated as yes/no/not applicable/cannot determine/not reported against each criterion. The risk of bias of a study was considered good if more than 80% of the answers were yes, fair if 50%–70% were yes, and poor if less than 50% were yes. Two coders were responsible for rating the risk of bias of the studies independently. Inconsistencies in risk of bias rating were resolved through discussion if necessary.

## Results

### Search Results

**Figure 1.**
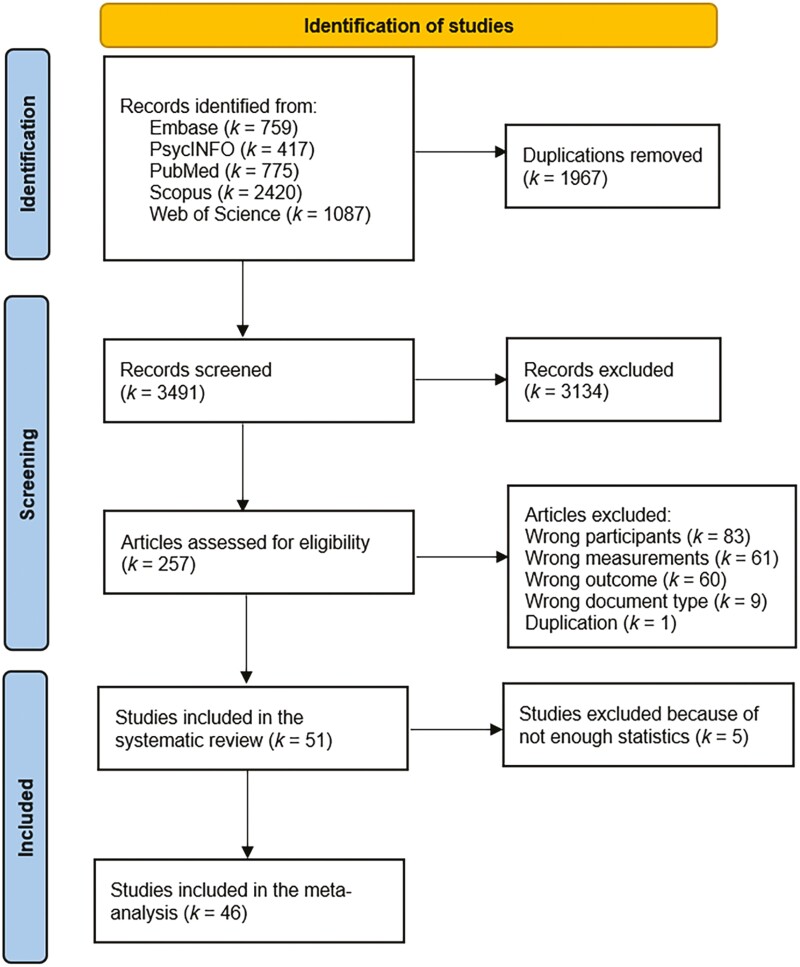
PRISMA flow chart of the study selection process.

The initial literature search yielded 5,458 publications by November 13, 2023. After removing duplicates, 3,491 publications remained. Following abstract screening, 257 publications were deemed suitable for full-text screening. Of these, 51 independent studies were included in the systematic review (Figure 1).

### Qualitative Synthesis

#### Participants

Forty-one studies were conducted in Western countries, seven studies were conducted in Asian or African countries, and three studies were conducted in multiple countries. In sum, 68,501 younger adults and 109,911 older adults were included in this systematic review.

#### Measures of prosociality

Prosociality was assessed with two categories of measurements, namely behavioral tasks and self-report scales (Table 1, data extraction was given in Supplementary Material 1). Researchers adopted different measurements to assess different types of prosociality. For sharing, the primary means for measurement is through behavioral tasks including monetary donation, dictator game, and other money-related tasks. For helping, the assessments varied significantly. Some participants were required to rate their willingness to help a person in need in an imaginary scenario (Ryan et al., 2022, 2023), whereas some were asked to finish additional tasks after the initial tasks (Bailey et al., 2020). For comforting, a group discussion process was coded by the assessors, which operationalized comforting as verbal sympathetic and empathetic utterances (Lakin et al., 1984). Self-report scales usually include multiple items to measure mixed types of prosociality (Cho et al., 2022; Jin et al., 2021).

**Table 1.** Summary Information of the Studies

Study	Country	*N*_younger adults	*N*_older adults	Measure of prosociality	Type
Albouy, 2018	France	170	173	Donation	Sharing
Bailey, 2013	Australia	35	34	Dictator game	Sharing
Bailey, 2020	Australia	40	39	Compiling the pamphlet	Helping
Beadle, 2015	United States	24	24	Dictator game	Sharing
Best, 2021, Study 2	Switzerland	48	53	Time donation	Helping
Bruine de Bruin, 2021	United States	498	693	Donation	Sharing
Byrne, 2023, Study 1	United States	65	46	Dictator game	Sharing
Byrne, 2023, Study 2	United States	42	38	Dictator game	Sharing
Chapman, 2023	Multiple	1186	108	Donation	Sharing
Cho, 2022	United States	204	69	Prosocial COVID-19 behaviors	Mixed
Chi et al., 2023	United States	134	31	Helping at work	Helping
Cutler, Wittmann et al., 2021	United Kingdom	75	77	Probabilistic reinforcement-learning task	Sharing
Cutler, Nitschke et al., 2021	Multiple	17,972	9115	Donation	Sharing
Dury, 2023	Belgium	858	155	Questionnaire	Mixed
Freund, 2014	Switzerland	37	37	Donation	Sharing
Gaesser, 2017	United States	31	39	Willingness to help when imagining scenarios	Helping
Gingras, 2021	Canada	40	40	Time spent for patients	Helping
Gong et al., 2017	Hong Kong	89	66	Donation	Sharing
Gunderson, 2022	United States	61	58	Donation	Sharing
Jin, 2021	Multiple	14,260	4696	Prosocial COVID-19 behaviors	Helping
Hao, 2016	China	62	49	Helping request	Helping
Kettner, 2016	Germany	272	167	Dictator game	Sharing
Lakin et al., 1984	United States	46	85	Group process behaviors	Comforting
Lockwood, 2021	United Kingdom	95	92	Physical-effort-based prosocial motivation	Sharing
Maxfield, 2014	United States	73	82	Prosocial preference	Mixed
Midlarsky, 1989, Study 1	United States	308	294	Donation	Sharing
Midlarsky, 1989, Study 2	United States	302	293	Donation	Sharing
Mõttus, 2015	Estonia	862	546	Altruism subscale	Mixed
Nakavachara, 2018	Thailand	28,821	90,886	Altruism questions from a survey	Mixed
Nava, 2023	Italy	163	89	Dictator game	Sharing
Ogawa, 2020	Japan	15	115	Dictator game	Sharing
Ojha, 2014	India	120	120	Altruism questionnaire	Mixed
Pokorski et al., 2013	Poland	59	61	Questionnaire of Altruism and Nonaltruism	Mixed
Pollerhoff et al., 2022	United States	71	62	A prosocial act question	Mixed
Pornpattananangkul, 2019	Singapore	39	39	Social-discounting task	Sharing
Rieger, 2015	Morocco	39	225	Dictator game	Sharing
Roalf, 2012	United States	29	30	Dictator game	Sharing
Roberts, 2019	United States	41	44	Donation	Sharing
Rosi, 2019	Italy	48	48	Dictator game	Sharing
Romano, 2021	Austria	130	118	Dictator game	Sharing
Ryan, 2022, Study 1	United States	100	100	Willingness to help when imagining scenarios	Helping
Ryan, 2022, Study 2	United States	96	93	Willingness to help when imagining scenarios	Helping
Ryan, 2022, Study 3	United States	95	102	Willingness to help when imagining scenarios	Helping
Ryan, 2023	Canada	83	91	Willingness to help when imagining scenarios	Helping
Sawczak, 2019	United States	28	34	Willingness to help when imagining scenarios	Helping
Sin, 2021	Canada, United States	424	262	COVID-19 related prosocial activity	Mixed
Sparrow, 2018, Study 1	Canada	32	30	Intertemporal choice task	Sharing
Sparrow, 2018, Study 2	Canada	31	23	Intertemporal choice task	Sharing
Sparrow, 2019	Canada	36	36	Intertemporal choice task	Sharing
Sze, 2012	United States	71	70	Donation	Sharing
Zak, 2022	United States	41	34	Donation	Sharing

*Notes*: COVID-19 = coronavirus disease 2019; *N*_younger adults = sample size of the younger adults; *N*_older adults = sample size of the older adults.

### Risk of Bias Assessment

Based on the Quality Assessment Tool for Observational Cohort and Cross-Sectional Studies, 32 studies (63%) were rated as good quality, 15 studies (29%) fair, and 4 studies (8%) poor (risk of bias assessment was given in Supplementary Material 2). The risks of bias mainly came from no justification for the sample size (*k* = 31), lack of clear inclusion/exclusion criteria of participants or recruiting participants from different populations (*k* = 21), failure to control key confounding variables such as social desirability and SES (*k* = 20), and unclear description of the study population (*k* = 5).

### Meta-Analysis

#### Age difference in prosociality

**Figure 2.**
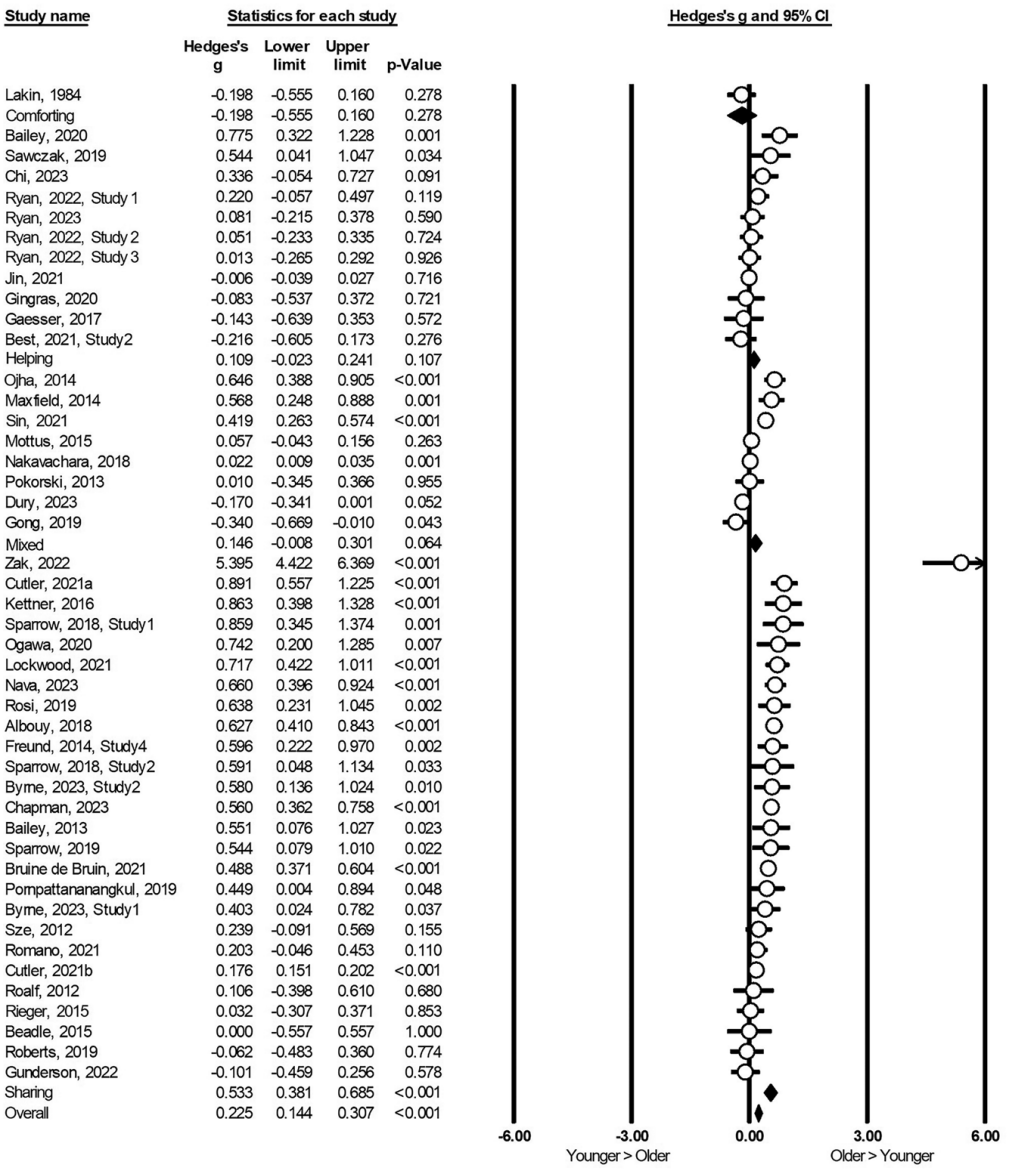
Forest plot of individual and summary effect size. CI = confidence interval.

The current meta-analysis included 46 independent studies with 109,144 older adults compared to 67,554 younger adults. Older adults were more prosocial than younger adults with a small to moderate effect size, Hedge’s *g* = 0.31, 95% confidence interval [0.24, 0.38] (Figure 2). There was a significant high heterogeneity among these 46 studies, *Q* (45) = 561.92, *p < *.001, *I*^2^ = 91.99%, τ = 0.17, τ^2^ = 0.03, supporting our examination of potential moderators of the overall effect.

#### Moderator analyses

Moderator analyses were conducted to assess the demographic and methodological factors that might moderate the age difference in prosociality (Table 2). Subgroup analyses were performed to examine the categorical moderators and meta-regressions were run on continuous moderators. The results suggested that the age effect was not moderated by education level, *Q* (2) = 3.05, *p* = .22, or whether the study was conducted in a Western country, non-Western country, or multiple countries, *Q* (2) = 2.89, *p* = .24. However, it was moderated by the SES of the older and younger adults, *Q* (2) = 8.46, *p* = .02. There was a significant age difference if the older adults had higher SES than younger adults (Hedges’ *g* = 0.41), whereas the age effect was not significant if the older adults had similar (Hedges’ *g* = 0.25) or lower SES (Hedges’ *g* = −0.20).

**Table 2.** Univariate Moderation Tests of the Variables for the Relationship Between Age and Prosociality

Moderator	Hedge’s *g*/*B*^a^	95% CI	*z*	*p* Value	*k*	Q
Demographic variables						
Mean age_younger adults	−0.02	[−0.06, 0.01]	−1.48	.14	35	2.18
Mean age_older adults	−0.01	[−0.04, 0.02]	−0.59	.56	35	0.35
Education				.22	16	3.05
Higher	0.24	[−0.05, 0.53]	1.60	.11	5	
Similar	0.53	[0.29, 0.77]	4.38	<.01	5	
Lower	0.22	[−0.21, 0.64]	0.99	.32	6	
Socioeconomic status				.02	14	8.46^*^
Higher	0.41	[0.21, 0.61]	4.00	<.01	9	
Similar	0.25	[−0.21, 0.70]	1.07	.29	4	
Lower	−0.20	[−0.56, 0.16]	−1.08	.28	1	
Country				.24	46	2.89
Western	0.38	[0.25, 0.51]	5.59	<.01	37	
Non-western	0.23	[−0.06, 0.52]	1.53	.13	6	
Multiple	0.20	[0.03, 0.38]	2.35	.02	3	
Methodological variables						
Type of prosociality				<.001	46	25.01^***^
Sharing	0.53	[0.38, 0.69]	6.87	<.01	26	
Helping	0.11	[−0.02, 0.24]	1.61	.11	11	
Comforting	−0.20	[−0.56, 0.16]	−1.08	.28	1	
Mixed	0.15	[−0.01, 0.30]	1.86	.06	7	
Measure				.02	46	5.45^*^
Behavior	0.43	[0.28, 0.58]	5.46	<.01	27	
Self-report	0.22	[0.14, 0.30]	5.35	<.01	19	

*Notes*: 95% CI = 95% confidence interval; *k* = number of independent studies; *p* = probability; *Q* = *Q* statistic for test of moderators; *z* = *z* test statistic.

^a^Hedge’s *g* is used for categorical moderators, whereas *B* is used for continuous moderators.

^*^ *p* < .05. ^***^p < .001.

The age effect was also moderated by the types of prosociality, *Q* (3) = 25.01, *p* < .001. Older adults were significantly more prosocial in sharing (Hedges’ *g* = 0.53), but not in helping (Hedges’ *g* = 0.11), comforting (Hedges’ *g* = −0.20), or mixed types of prosociality (Hedges’ *g* = 0.15). The age effect was also moderated by whether the prosociality was measured through behavioral tasks or self-report scales, *Q* (1) = 5.45, *p* = .02. The age difference was larger in behavioral tasks (Hedges’ *g* = 0.43) compared to self-report scales (Hedges’ *g* = 0.22). However, the moderation effect of measure was not significant after controlling for the type of prosociality (*p* = .50), whereas the moderation effect of type was still significant after controlling for the measure of prosociality (*p* = .001). It was suggested that the moderation effect was mainly due to types of prosociality.

#### Publication bias

**Figure 3.**
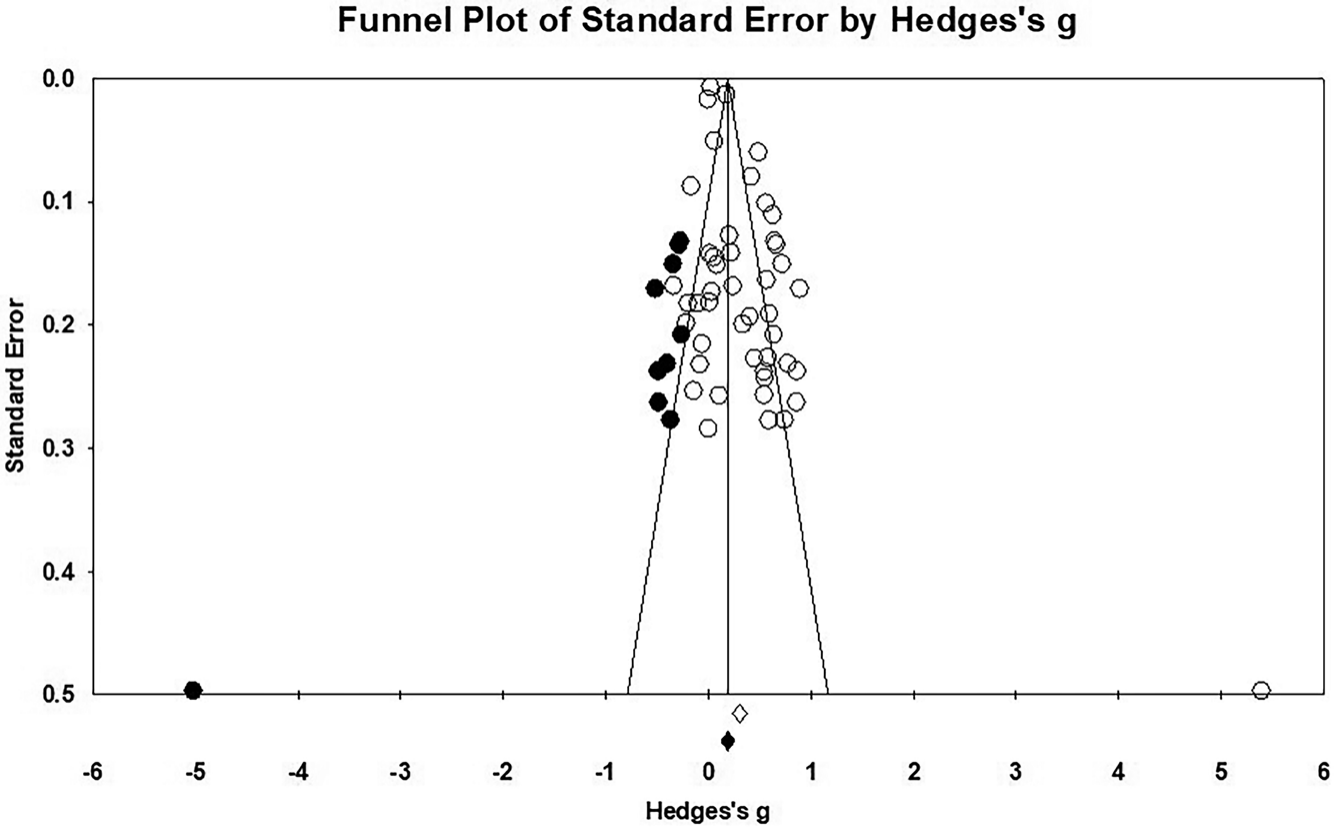
Funnel plot examining the publication bias. Imputed point estimated from the trim-and-fill analysis were shaded in black.

The funnel plot of effect sizes seemed to be roughly symmetrical (Figure 3). The classical fail-safe *N* analysis was performed to check for publication bias, *z* = 16.38, *p* < .001, *N* = 3,167, indicating that the number of studies required for a nonsignificant effect size was more than 68 times the number of the included studies. The trim-and-fill analysis was conducted to adjust the potential publication bias, and 10 studies were imputed. Egger’s regression intercept yielded a significant bias intercept, β = 2.04, *t*(44) = 4.06, *p < *.001. To summarize, the three approaches used to examine publication bias suggested some potential bias in our data.

#### Quality of evidence

Based on the risk of bias assessment of each study, high inconsistency across the studies, and moderate publication bias, the estimate of effect can be interpreted with moderate confidence.

## Discussion

The current review comprehensively summarized 51 studies on aging and different types of prosociality. Although older adults are generally more prosocial than younger adults, there is great heterogeneity across the studies, which can be explained by the type of prosociality, the SES difference between older and younger adults, and the measure of prosociality.

### Age Difference in Prosociality

The current study found that older adults were more prosocial in general, which was consistent with existing reviews on prosociality and aging. Although researchers seem to agree that older adults act more prosocially than younger adults, different theories have been proposed to explain this age difference and each of them is supported by some studies included in the current review. However, the existing theories considered prosociality as a single-dimensional construct and did not look into different types of prosociality separately. We have advanced the theory by providing more nuanced understanding of the relationship between aging and prosociality. Based on the results of the current review, we highlight some important moderators that could influence the age difference in prosociality.

### Moderators Between Age and Prosociality

#### Older adults were more prosocial than younger adults in sharing than other types of prosociality

The current meta-analysis found that the types of prosocial behavior had a significant moderating effect on the relationship between age and prosociality. Although older adults were more prosocial when sharing money or materials, they might not be so when helping or comforting others. This might be attributable to their perspectives of resources and motivations. According to the Value-based Decision Framework, people hold different resources that could support diverse types of prosocial behavior (Mayr & Freund, 2020). As older adults have more financial resources (Huggett, 1996) and annual income (Roalf et al., 2012; Roberts & Maxfield, 2019), they consider the cost of sharing as little and tend to donate more money as compared to younger adults. Contrarily, older adults have limited time resources, so a time-consuming helping task could refrain them from giving a hand. This explains why older and younger adults have a similar tendency to assist others if they have to wait before actually helping a patient in a prosocial behavior paradigm where participants acted as a healthcare professional and determined how much time to spend helping patients (Gingras et al., 2021). In the case of comforting others, studies suggested that older adults had fewer cognitive resources for recognizing others’ emotions simultaneously (Henry et al., 2013; Osterhaus & Bosacki, 2022), which prevented them from comforting others during a group discussion process.

On the other hand, older adults have stronger prosocial motivation as claimed by the general benevolence model (Hubbard et al., 2016). Specifically, the age difference in prosocial sharing was still significant after controlling for the influence of SES (Beadle et al., 2015), which provided robust evidence that wealth was not the only driver of sharing. Furthermore, some studies found that older adults were more prosocial even in money-irrelevant tasks, such as helping with various situations (Sawczak et al., 2019). However, other research suggested that older adults were less prosocial when comforting others, which was at odds with their generally more prosocial orientation. This might be explained by the Strength and Vulnerability Integration theory (Charles, 2010) that posits older adults tend to avoid exposure to negative emotional events. Because older adults had reduced flexibility of biological systems, as evidenced by greater fluctuations in blood pressure and prolonged hemodynamic response, they would avoid negative experiences in the first place to maintain emotional well-being. A meta-analysis on the age-related positivity effect also indicated that older adults preferred positive information to negative information, whereas younger adults exhibited the opposite preference (Reed et al., 2014). To summarize, existing research suggested that older adults were less likely to comfort others as compared to younger adults.

#### Age difference in prosociality was larger when older adults had higher SES than younger adults

The significant moderating effect of SES could be explained by the Value-based Decision Framework (Mayr & Freund, 2020). Pursuant to which, financial condition is a vital constraint of prosocial behavior. SES was operationalized as income in the included studies. In general, older adults possess more financial resources, whereas younger adults, especially university students, had limited financial resources. It should be noted that a few studies recruited younger and older participants from different sources (Bailey et al., 2013; Beadle et al., 2015; Maxfield et al., 2014; Sawczak et al., 2019). In these studies, the younger adults comprised university students, whereas the older adults were community-dwelling volunteers. As the younger adults could be participating for utilitarian purposes (receiving course credits or money) and the older adults could be there for altruistic purposes (volunteering), the sampling bias further increased the SES difference and could lead to the age difference in prosociality.

An emerging question is whether SES affects prosociality. Although our results suggested a positive association between SES and prosociality, this appeared to be contradictory to some of the previous studies. For instance, some research on younger adults found that individuals with higher SES were less generous in a dictator game, less charitable in donation, less trusting in a trust game, less helpful in a cooperative task (Piff et al., 2010), and less compassionate towards others’ suffering (Stellar et al., 2012). Similarly, Hui, Ngai, et al. (2020) reported that lower SES was associated with more informal helping and more volunteering among youth. On the other hand, some studies do concur with our findings. For instance, Korndörfer et al. (2015) conducted a large-scale test on the effect of SES on prosocial behavior and the results indicated that people with higher SES made more charitable donations, helped more in everyday situations, and volunteered more. However, it should be noted that these studies focused primarily on younger adults, especially undergraduate students. In other words, they offered little evidence for the relationship between SES and prosociality in older adults. Li and Siu (2021) examined the relationship among SES, empathic concern and prosocial tendency in a continuous range of ages. Its results supported that SES significantly moderated the age difference in empathic concern, that is, being older was associated with more empathy only when participants had high SES and the association was not significant when participants had low SES. This age-related difference in empathic concern in turn led to divergent prosocial tendencies, suggesting that SES also influenced prosociality indirectly through empathy. Overall, more research is needed to clarify the relationship between SES and prosociality, particularly among older adults.

#### Age difference in prosociality was larger in behavioral tasks than in self-report scales

The significant moderation effect of measure of prosociality might be related to two reasons. First, older adults might have different interpretations of the item wordings compared to younger adults and these interpretations further contribute to shifted responses on self-report scales. A study summarized the potential sources of response bias in self-report scales with older adults, including complicated instructions, vague meaning in question wording, vague response categories, and memory bias (Hill et al., 2019). For example, the Self-Report Altruism scale (Rushton et al., 1981) asks participants to recall if they helped others in the past month. However, the instructions of behavioral tasks can be relatively simple and easy to understand, for example, asking participants if they can donate money at the moment (Midlarsky & Hannah, 1989). Second, some self-report measures need imagination and older adults may have less vividness during imagination, thus leading to less self-report prosociality. For example, Gaesser et al. (2017) developed a series of scenarios where people need help and asked participants to imagine the scenarios and rate the likelihood of helping. To summarize, behavioral tasks might capture more naturalistic responses than self-report scales from participants, especially older participants.

#### Age difference in prosociality was significant in Western countries but not in non-Western countries

Our results suggested that there might be cultural difference in the age difference in prosociality. The age difference was only significant in Western countries or multiple countries, whereas it was not significant in non-Western countries (e.g., China) according to the results of subgroup analyses. The role of cultural norms should be noted in the lifespan development of prosociaity, especially costly prosociality. For example, a study asked American children (aged from 3 to 14 years) and Fijian children to play a dictator game and found different trajectories across the two groups (House et al., 2013), indicating social norms shape the ontogeny of prosociality in childhood. This current meta-analysis provides evidence that the influence of social norms on prosociality might continue into old age.

### Limitations

There are several limitations to this meta-analysis. First, the number of studies included in the subgroup analyses was limited according to the standard of the Cochrane Handbook, which recommended at least 10 studies for each group (Higgins et al., 2019). There was only one study on comforting in the current review, so our findings should be interpreted cautiously. More studies on comforting in older adults are needed to corroborate the findings of our research. Additionally, non-English studies were not included in the current review, so the cultural difference of development of prosociality remains to be investigated.

Second, mediators between aging and prosociality cannot be examined in this meta-analysis. Some studies investigated possible mediators such as empathy (Cutler, Wittmann, et al., 2021), reasoning skills (Rosi et al., 2019), and the behavioral tendency of trusting instead of suspecting others (Gunderson & ten Brinke, 2022). However, data on mediators were lacking in many studies, which limited our ability to conclude the underlying mechanisms that linked aging and prosociality.

Third, the neural mechanisms of prosocial behavior in older adults remain unclear due to the limited number of studies. To date, there is only one functional magnetic resonance imaging study that compared brain activation in older adults and younger adults during an ultimatum game. It was found that older adults showed higher activation in the dorsolateral prefrontal cortex compared to younger adults, suggesting that older adults might rely more on brain regions supporting computation to make economic decisions (Harlé & Sanfey, 2012). However, because ultimatum game might not reflect prosocial behavior (Yamagishi et al., 2012), the results are insufficient for concluding the neural basis of prosociality.

### Future Directions

The current systematic review and meta-analysis provided several insights and suggestions for future research. First, more reliable, valid, and comprehensive measures of prosocial behavior need to be developed. Currently, most studies measure prosociality using money-related tasks because money can be easily quantified and compared between different populations. However, this approach is not comprehensive because only sharing is measured. Besides, the reliability and validity of these measurements might be questionable. For example, the test-retest reliability of a one-shot hypothetical dictator game is low because participants already know that there is no confederate to share money with (Kettner & Waichman, 2016). Thus, a more comprehensive tool with good psychometric properties is required to fully examine prosocial behavior.

Second, more research is needed to identify moderators and mediators that may influence the age-related changes in prosociality. One potential moderator is in-group preference. A recent study found that older adults generally had a stronger in-group preference, which led to more donations to national charities and fewer donations to international charities as compared to younger adults (Cutler, Nitschke, et al., 2021). However, another study revealed that older adults had a weaker in-group preference, which prompted them to donate more to socially distant others (Pornpattananangkul et al., 2019). Although previous research has provided valuable information on the age-related changes in prosociality, there is still much to be learned about the factors that influence these differences.

Third, future research could look into the neural correlates of age-related changes in prosociality. Past studies on empathy suggested that older adults showed greater activation in brain areas associated with empathy (anterior cingulate cortex and insula) when they witnessed others in distress (Ziaei et al., 2021). Given the close relationship between empathy and prosociality, future studies may investigate the neural underpinnings of prosociality. This line of research may provide insights into the mechanisms of prosociality and potential interventions to promote prosocial behavior.

## Conclusion

The current systematic review and meta-analysis summarized the mixed findings on healthy aging and prosocial behavior. In general, older adults are more prosocial than younger adults, but the age effect is moderated by the types of prosociality and SES. Future research should develop more valid assessment tools and utilize neuroimaging techniques to comprehensively understand the relationship between aging and prosocial behavior.

## Supplementary Material

gnae082_suppl_Supplementary_Material

## Data Availability

Data extracted from included studies are available in the Supplementary Materials. This review was preregistered at PROSPERO (CRD42022333373).
