# Supplementary material for: Are Older Adults More Prosocial Than Younger Adults? A Systematic Review and Meta-Analysis
Source: Gerontologist. 2024 Jul 4;64(9):gnae082. doi: 10.1093/geront/gnae082 (PMC11322677; doi:10.1093/geront/gnae082)
Supplement: gnae082_suppl_Supplementary_Material [file gnae082_suppl_supplementary_material.docx]

**Supplementary Material**

**Supplementary Material 1.** PRISMA Checklist

| **Section and Topic** | **Item #** | **Checklist item** | **Location where item is reported** |
| --- | --- | --- | --- |
| **TITLE** | | |  |
| Title | 1 | Identify the report as a systematic review. | Page 1 |
| **ABSTRACT** | | |  |
| Abstract | 2 | See the PRISMA 2020 for Abstracts checklist. | Page 3 |
| **INTRODUCTION** | | |  |
| Rationale | 3 | Describe the rationale for the review in the context of existing knowledge. | Page 8 |
| Objectives | 4 | Provide an explicit statement of the objective(s) or question(s) the review addresses. | Page 8 |
| **METHODS** | | |  |
| Eligibility criteria | 5 | Specify the inclusion and exclusion criteria for the review and how studies were grouped for the syntheses. | Page 9 |
| Information sources | 6 | Specify all databases, registers, websites, organisations, reference lists and other sources searched or consulted to identify studies. Specify the date when each source was last searched or consulted. | Page 9 |
| Search strategy | 7 | Present the full search strategies for all databases, registers and websites, including any filters and limits used. | Page 9 |
| Selection process | 8 | Specify the methods used to decide whether a study met the inclusion criteria of the review, including how many reviewers screened each record and each report retrieved, whether they worked independently, and if applicable, details of automation tools used in the process. | Page 9 |
| Data collection process | 9 | Specify the methods used to collect data from reports, including how many reviewers collected data from each report, whether they worked independently, any processes for obtaining or confirming data from study investigators, and if applicable, details of automation tools used in the process. | Page 10 |
| Data items | 10a | List and define all outcomes for which data were sought. Specify whether all results that were compatible with each outcome domain in each study were sought (e.g. for all measures, time points, analyses), and if not, the methods used to decide which results to collect. | Page 10 |
|  | 10b | List and define all other variables for which data were sought (e.g. participant and intervention characteristics, funding sources). Describe any assumptions made about any missing or unclear information. | Page 10 |
| Study risk of bias assessment | 11 | Specify the methods used to assess risk of bias in the included studies, including details of the tool(s) used, how many reviewers assessed each study and whether they worked independently, and if applicable, details of automation tools used in the process. | Page 11 |
| Effect measures | 12 | Specify for each outcome the effect measure(s) (e.g. risk ratio, mean difference) used in the synthesis or presentation of results. | Page 10 |
| Synthesis methods | 13a | Describe the processes used to decide which studies were eligible for each synthesis (e.g. tabulating the study intervention characteristics and comparing against the planned groups for each synthesis (item #5)). | Page 9 |
|  | 13b | Describe any methods required to prepare the data for presentation or synthesis, such as handling of missing summary statistics, or data conversions. | Page 10 |
|  | 13c | Describe any methods used to tabulate or visually display results of individual studies and syntheses. | Page 10 |
|  | 13d | Describe any methods used to synthesize results and provide a rationale for the choice(s). If meta-analysis was performed, describe the model(s), method(s) to identify the presence and extent of statistical heterogeneity, and software package(s) used. | Page 10 |
|  | 13e | Describe any methods used to explore possible causes of heterogeneity among study results (e.g. subgroup analysis, meta-regression). | Page 10 |
|  | 13f | Describe any sensitivity analyses conducted to assess robustness of the synthesized results. | Page 10 |
| Reporting bias assessment | 14 | Describe any methods used to assess risk of bias due to missing results in a synthesis (arising from reporting biases). | Page 11 |
| Certainty assessment | 15 | Describe any methods used to assess certainty (or confidence) in the body of evidence for an outcome. | Page 13 |
| **RESULTS** | | |  |
| Study selection | 16a | Describe the results of the search and selection process, from the number of records identified in the search to the number of studies included in the review, ideally using a flow diagram. | Page 11 |
|  | 16b | Cite studies that might appear to meet the inclusion criteria, but which were excluded, and explain why they were excluded. | Page 9 |
| Study characteristics | 17 | Cite each included study and present its characteristics. | Page 35 |
| Risk of bias in studies | 18 | Present assessments of risk of bias for each included study. | Page 13 |
| Results of individual studies | 19 | For all outcomes, present, for each study: (a) summary statistics for each group (where appropriate) and (b) an effect estimate and its precision (e.g. confidence/credible interval), ideally using structured tables or plots. | Page 39 |
| Results of syntheses | 20a | For each synthesis, briefly summarise the characteristics and risk of bias among contributing studies. | Page 12 |
|  | 20b | Present results of all statistical syntheses conducted. If meta-analysis was done, present for each the summary estimate and its precision (e.g. confidence/credible interval) and measures of statistical heterogeneity. If comparing groups, describe the direction of the effect. | Page 14 |
|  | 20c | Present results of all investigations of possible causes of heterogeneity among study results. | Page 14 |
|  | 20d | Present results of all sensitivity analyses conducted to assess the robustness of the synthesized results. | Page 14 |
| Reporting biases | 21 | Present assessments of risk of bias due to missing results (arising from reporting biases) for each synthesis assessed. | Page 15 |
| Certainty of evidence | 22 | Present assessments of certainty (or confidence) in the body of evidence for each outcome assessed. | Page 15 |
| **DISCUSSION** | | |  |
| Discussion | 23a | Provide a general interpretation of the results in the context of other evidence. | Page 15 |
|  | 23b | Discuss any limitations of the evidence included in the review. | Page 20 |
|  | 23c | Discuss any limitations of the review processes used. | Page 20 |
|  | 23d | Discuss implications of the results for practice, policy, and future research. | Page 21 |
| **OTHER INFORMATION** | | |  |
| Registration and protocol | 24a | Provide registration information for the review, including register name and registration number, or state that the review was not registered. | Page 3 |
|  | 24b | Indicate where the review protocol can be accessed, or state that a protocol was not prepared. | Page 3 |
|  | 24c | Describe and explain any amendments to information provided at registration or in the protocol. | NA |
| Support | 25 | Describe sources of financial or non-financial support for the review, and the role of the funders or sponsors in the review. | Page 1 |
| Competing interests | 26 | Declare any competing interests of review authors. | Page 1 |
| Availability of data, code and other materials | 27 | Report which of the following are publicly available and where they can be found: template data collection forms; data extracted from included studies; data used for all analyses; analytic code; any other materials used in the review. | Page 1 |

**Supplementary Material 2.** Data Extraction.

| **Study** | **Conditions** | **Mean older adults** | **SD older adults** | **N older adults** | **Mean younger adults** | **SD younger adults** | **N younger adults** | **Cohen's d** | **p value** | **Mean age younger adults** | **Mean age older adults** | **P male younger adults** | **P male older adults** | **Education (Older adults compared to younger adults)** | **SES**  **(Older adults compared to younger adults)** | **Country** | **Country (Western/**  **Non-western/ Multiple)** | **Type of prosociality** | **Self-report/**  **Behavior** | **No. of items** | **Quality** |
| --- | --- | --- | --- | --- | --- | --- | --- | --- | --- | --- | --- | --- | --- | --- | --- | --- | --- | --- | --- | --- | --- |
| Albouy, 2018 | weak | 4.875 | 1.363 | 173 | 3.758 | 1.763 | 170 |  |  | - | - | 0.440 | 0.410 | - | - | France | Western | share | Self-report | 3 | fair |
| Albouy, 2018 | moderate | 4.522 | 1.685 | 173 | 3.409 | 1.722 | 170 |  |  | - | - | 0.440 | 0.410 | - | - | France | Western | share | Self-report | 3 | fair |
| Albouy, 2018 | high | 4.677 | 1.573 | 173 | 3.879 | 1.484 | 170 |  |  | - | - | 0.440 | 0.410 | - | - | France | Western | share | Self-report | 3 | fair |
| Bailey, 2013 | overall | 5.88 | 1.91 | 34 | 5.03 | 1.02 | 35 |  |  | 21.3 | 73.9 | 0.486 | 0.294 | less | - | Australia | Western | share | Behavior | 20 | good |
| Bailey, 2020 | overall | 0.58 | 0.24 | 39 | 0.38 | 0.27 | 40 |  |  | 19.88 | 72.44 | 0.300 | 0.359 | same | - | Australia | Western | help | Behavior | 1 | good |
| Beadle, 2015 | overall | 3.79 | 1.86 | 24 | 3.79 | 1.61 | 24 |  |  | 19.83 | 77.92 | 0.375 | 0.375 | more | more | US | Western | share | Behavior | 1 | good |
| Best, 2021, Study2 | overall | 663.49 | 312.55 | 48 | 727.83 | 279.57 | 53 |  |  | 23.65 | 73.21 | - | - | - | - | Switzerland | Western | help | Behavior | 1 | fair |
| Bruine de Bruin, 2021 | overall | 3.81 | 1.96 | 693 | 2.82 | 2.12 | 498 |  |  | - | - | 0.430 | 0.430 | - | - | US | Western | share | Behavior | 1 | good |
| Byrne, 2023, Study1 | overall | 0.299 | 0.185 | 46 | 0.223 | 0.189 | 65 |  |  | 27.43 | 69.57 | 0.415 | 0.435 | - | more | US | Western | share | Behavior | 40 | good |
| Byrne, 2023, Study2 | overall | 0.34 | 0.191 | 38 | 0.216 | 0.229 | 42 |  |  | 25.07 | 69.71 | 0.429 | 0.289 | - | more | US | Western | share | Behavior | - | good |
| Chapman, 2023 | overall | 0.769 | 0.422 | 108 | 0.5 | 0.485 | 1186 |  |  | 24.84 | 65 | 0.184 | 0.435 | - | - | Multiple | Multiple | share | Self-report | 1 | fair |
| Chi, 2023 | overall | 0.455 | 0.254 | 31 | 0.365 | 0.269 | 134 |  |  | 30.1 | 63.1 | 0.351 | 0.548 | same | same | US | Western | help | Self-report | - | good |
| Cutler, 2021a | overall | 0.0177 | 0.0566 | 75 | -0.0364 | 0.064 | 75 |  |  | 23.07 | 69.84 | 0.410 | 0.480 | - | - | UK | Western | share | Behavior | 144 | good |
| Cutler, 2021b | overall | 8.705 | 1.679 | 9115 | 8.395 | 1.796 | 17972 |  |  | 26.81 | 67.02 | - | - | - | - | Multiple | Multiple | share | Self-report | 1 | fair |
| Dury, 2023 | overall |  |  | 155 |  |  | 858 |  | 0.026 | - | - | - | - | less | - | Belgium | Western | mixed | Self-report | 29 | fair |
| Freund, 2014, Study4 | overall | 0.594 | 0.491 | 69 | 0.306 | 0.464 | 48 |  |  | 24.1 | 68.8 | 0.500 | 0.391 | - | more | Switzerland | Western | share | Behavior | 1 | good |
| Gaesser, 2017 | overall | 5.02 | 1.169231 | 31 | 5.2 | 1.314534 | 30 |  |  | 21.97 | 74 | - | - | more | - | US | Western | help | Self-report | 3 | good |
| Gingras, 2020 | overall | 43.33 | 12.87 | 38 | 44.34 | 11.12 | 35 |  |  | 22.45 | 63.75 | 0.500 | 0.500 | - | - | Canada | Western | help | Behavior | 56 | good |
| Gong, 2019 | money nonrelative | 5.67 | 1.63 | 66 | 7.44 | 0.77 | 89 |  |  | 30.12 | 69.39 | 0.393 | 0.424 | less | same | Hong Kong | Non-western | mixed | Behavior | 28 | good |
| Gong, 2019 | money relative | 7.03 | 1.03 | 66 | 7.27 | 0.95 | 89 |  |  | 30.12 | 69.39 | 0.393 | 0.424 | less | same | Hong Kong | Non-western | mixed | Behavior | 28 | good |
| Gong, 2019 | time nonrelative | 5.71 | 1.54 | 66 | 6.14 | 1.41 | 89 |  |  | 30.12 | 69.39 | 0.393 | 0.424 | less | same | Hong Kong | Non-western | mixed | Behavior | 28 | good |
| Gong, 2019 | time relative | 7.02 | 1.11 | 66 | 6.29 | 1.19 | 89 |  |  | 30.12 | 69.39 | 0.393 | 0.424 | less | same | Hong Kong | Non-western | mixed | Behavior | 28 | good |
| Gunderson, 2022 | overall | 39.859 | 28.939 | 61 | 42.522 | 22.715 | 58 |  |  | 19.03 | 74.12 | 0.213 | 0.259 | - | - | US | Western | share | Behavior | 12 | good |
| Jin, 2021 | overall | 6.213 | 0.968 | 4696 | 6.219 | 0.986 | 14260 |  |  | - | - | 0.633 | 0.467 | - | - | Multiple | Multiple | help | Self-report | 4 | good |
| Kettner, 2016 | overall | 0.446 | 0.275 | 26 | 0.241 | 0.219 | 68 |  |  | 22.83 | 68.53 | - | - | - | more | Germany | Western | share | Behavior | 1 | fair |
| Lakin, 1984 | overall | 3.52 | 0.88 | 85 | 3.68 | 0.64 | 46 |  |  | 19.64 | 70.79 | 0.391 | 0.223 | - | less | US | Western | comfort | Behavior | 181 | poor |
| Lockwood, 2021 | overall | 349.38 | 9.42 | 92 | 300.02 | 95.76 | 95 |  |  | 24 | 69 | 0.410 | 0.530 | same | - | UK | Western | share | Behavior | 150 | good |
| Maxfield, 2014 | overall | 1.9 | 2.35 | 82 | 0.57 | 2.31 | 73 |  |  | 19.02 | 72 | 0.411 | 0.098 | - | - | US | Western | mixed | Self-report | 2 | fair |
| Mottus, 2015 | A1 | 2.550599 | 1.061053 | 716 | 2.228538 | 1.09161 | 862 |  |  | - | - | 0.465 | 0.462 | - | - | Estonia | Western | mixed | Self-report | 8 | fair |
| Mottus, 2015 | A2 | 3.273106 | 0.778183 | 716 | 3.178654 | 0.842048 | 862 |  |  | - | - | 0.465 | 0.462 | - | - | Estonia | Western | mixed | Self-report | 8 | fair |
| Mottus, 2015 | A3 | 2.43566 | 0.994793 | 716 | 2.278422 | 1.123696 | 862 |  |  | - | - | 0.465 | 0.462 | - | - | Estonia | Western | mixed | Self-report | 8 | fair |
| Mottus, 2015 | A4 | 3.303859 | 0.673176 | 716 | 3.2413 | 0.644651 | 862 |  |  | - | - | 0.465 | 0.462 | - | - | Estonia | Western | mixed | Self-report | 8 | fair |
| Mottus, 2015 | A5 | 2.168015 | 0.818652 | 716 | 2.363109 | 0.782612 | 862 |  |  | - | - | 0.465 | 0.462 | - | - | Estonia | Western | mixed | Self-report | 8 | fair |
| Mottus, 2015 | A6 | 2.522594 | 0.7805 | 716 | 2.684455 | 0.74054 | 862 |  |  | - | - | 0.465 | 0.462 | - | - | Estonia | Western | mixed | Self-report | 8 | fair |
| Mottus, 2015 | A7 | 2.794093 | 0.812587 | 716 | 2.966357 | 0.730952 | 862 |  |  | - | - | 0.465 | 0.462 | - | - | Estonia | Western | mixed | Self-report | 8 | fair |
| Mottus, 2015 | A8 | 3.05616 | 0.772693 | 716 | 2.651972 | 0.898826 | 862 |  |  | - | - | 0.465 | 0.462 | - | - | Estonia | Western | mixed | Self-report | 8 | fair |
| Nakavachara, 2018 | Q1 | 1.942 | 0.502 | 90886 | 1.921 | 0.512 | 28821 |  |  | - | - | - | - | - | - | Thailand | Non-western | mixed | Self-report | 3 | poor |
| Nakavachara, 2018 | Q2 | 1.98 | 0.506 | 90886 | 1.966 | 0.509 | 28821 |  |  | - | - | - | - | - | - | Thailand | Non-western | mixed | Self-report | 3 | poor |
| Nakavachara, 2018 | Q3 | 1.87 | 0.555 | 90886 | 1.872 | 0.551 | 28821 |  |  | - | - | - | - | - | - | Thailand | Non-western | mixed | Self-report | 3 | poor |
| Nava, 2023 | overall | 1.767 | 1.212 | 89 | 1.074 | 0.944 | 163 |  |  | 22.94 | 65.68 | 0.319 | 0.517 | less | - | Italy | Western | share | Behavior | 1 | good |
| Ogawa, 2020 | overall | 629.5652 | 437.0904 | 115 | 306.6667 | 391.8211 | 15 |  |  | - | - | - | - | - | - | Japan | Non-western | share | Behavior | 1 | fair |
| Ojha 2014 | overall | 23.74 | 7.23 | 120 | 19.1 | 7.08 | 120 |  |  | - | - | - | - | - | - | India | Non-western | mixed | Self-report | 20 | fair |
| Pokorski, 2013 | overall | 72.8 | 10.2 | 61 | 72.7 | 9 | 59 |  |  | 24 | 67 | 0.440 | 0.180 | - | - | Poland | Western | mixed | Self-report | 17 | fair |
| Pornpattananangkul, 2019 | overall | 0.4063 | 0.3159 | 39 | 0.2859 | 0.2025 | 39 |  |  | 22.79 | 69.69 | 0.359 | 0.462 | less | - | Singapore | Non-western | share | Behavior | 72 | good |
| Rieger, 2015 | overall | 2.031 | 1.255 | 225 | 1.991 | 1.199 | 39 |  |  | 24.513 | 71.267 | 0.923 | 0.942 | - | - | Morocco | Non-western | share | Self-report | 3 | good |
| Roalf, 2012 | overall | 4.23 | 1.7 | 30 | 4 | 2.51 | 29 |  |  | - | - | 0.517 | 0.500 | same | more | US | Western | share | Behavior | 1 | good |
| Roberts, 2019 | overall | 2.25 | 4.34 | 44 | 2.52 | 4.34 | 41 |  |  | 25.17 | 71.95 | - | - | more | more | US | Western | share | Behavior | 2 | fair |
| Romano, 2021 | Younger partners | 5.03 | 3.23 | 118 | 4.16 | 2.69 | 130 |  |  | 28 | 69.7 | 0.423 | 0.458 | - | - | Austria | Western | share | Behavior | 1 | good |
| Romano, 2021 | Middle-aged parteners | 3.84 | 3.01 | 118 | 3.74 | 2.46 | 130 |  |  | 28 | 69.7 | 0.423 | 0.458 | - | - | Austria | Western | share | Behavior | 1 | good |
| Romano, 2021 | Old parteners | 5.17 | 3.43 | 118 | 4.28 | 2.89 | 130 |  |  | 28 | 69.7 | 0.423 | 0.458 | - | - | Austria | Western | share | Behavior | 1 | good |
| Rosi, 2019 | overall | 3.33 | 0.72 | 48 | 2.87 | 0.71 | 48 |  |  | 23.29 | 70.19 | 0.625 | 0.792 | - | - | Italy | Western | share | Behavior | 8 | fair |
| Ryan, 2022, Study 1 | overall | 4.94 | 1.9 | 100 | 4.52 | 1.9 | 100 |  |  | 28.05 | 67 | 0.39 | 0.49 | - | - | US | Western | help | Self-report | 6 | good |
| Ryan, 2022, Study 2 | overall | 5.09 | 2.12 | 93 | 4.98 | 2.156 | 96 |  |  | 28.17 | 66.62 | 0.354 | 0.462 | - | - | US | Western | help | Self-report | 6 | good |
| Ryan, 2022, Study 3 | overall | 5 | 2.24 | 102 | 4.97 | 2.32 | 95 |  |  | 27.24 | 66.51 | 0.242 | 0.402 | - | - | US | Western | help | Self-report | 6 | good |
| Ryan, 2023 | overall | 5.16 | 1.79 | 91 | 5.01 | 1.88 | 83 |  |  | 22.88 | 70.88 | 0.349 | 0.462 | - | - | Canada | Western | help | Self-report | 6 | good |
| Sawczak, 2019 | overall | 5.78 | 0.97 | 34 | 5.21 | 1.11 | 28 |  |  | 19 | 73.2 | 0.286 | 0.324 | - | - | US | Western | help | Self-report | 8 | good |
| Sin, 2021 | overall | 0.38 | 0.34 | 262 | 0.25 | 0.29 | 424 |  |  | - | - | 0.094 | 0.168 | - | - | Canada US | Western | mixed | Self-report | 1 | fair |
| Sparrow, 2018, Study1 | overall |  |  | 30 |  |  | 32 | 0.87 |  | 25.28 | 70.57 | 0.380 | 0.370 | more | same | Canada | Western | share | Behavior | 84 | good |
| Sparrow, 2018, Study2 | overall |  |  | 23 |  |  | 31 | 0.6 |  | 20.84 | 71.35 | 0.390 | 0.390 | same | more | Canada | Western | share | Behavior | 84 | good |
| Sparrow, 2019 | overall |  |  | 36 |  |  | 36 | 0.55 |  | 21 | 70.11 | 0.420 | 0.370 | more | more | Canada | Western | share | Behavior | 84 | good |
| Sze, 2012 | uplifting | 1.74 | 2.91 | 70 | 1.46 | 2.09 | 71 |  |  | 23.07 | 66.43 | - | - | more | same | US | Western | share | Behavior | 4 | good |
| Sze, 2012 | distressing | 3.84 | 3.81 | 70 | 2.63 | 2.63 | 71 |  |  | 23.07 | 66.43 | - | - | more | same | US | Western | share | Behavior | 4 | good |
| Zak, 2022 | overall | 12.45 | 1.79 | 34 | 4.64 | 1.05 | 41 |  |  | - | - | - | - | - | - | US | Western | share | Behavior | 1 | good |

*Note.* SES = Socioeconomic status.

**Supplementary Material 3.** Risk of Bias Assessment.

| Study | 1. Was the research question or objective in this paper clearly stated? | 2. Was the study population clearly specified and defined? | 3. Were all the subjects selected or recruited from the same or similar populations (including the same time period)? Were inclusion and exclusion criteria for being in the study prespecified and applied uniformly to all participants? | 4. Was a sample size justification, power description, or variance and effect estimates provided? | 5. For the analyses in this paper, were the exposure(s) of interest measured prior to the outcome(s) being measured? | 6. For exposures that can vary in amount or level, did the study examine different levels of the exposure as related to the outcome (e.g., categories of exposure, or exposure measured as continuous variable)? | 7. Were the exposure measures clearly defined, valid, reliable, and implemented consistently across all study participants? | 8. Was the exposure(s) assessed more than once over time? | 9. Were the outcome measures (dependent variables) clearly defined, valid, reliable, and implemented consistently across all study participants? | 10. Were key potential confounding variables measured and adjusted statistically for their impact on the relationship between exposure(s) and outcome(s)? | Quality Rating |
| --- | --- | --- | --- | --- | --- | --- | --- | --- | --- | --- | --- |
| Albouy, 2018 | Yes | No | No | No | Yes | Yes | Yes | Yes | Yes | No | Fair |
| Bailey, 2013 | Yes | Yes | No | No | Yes | Yes | Yes | Yes | Yes | Yes | Good |
| Bailey, 2020 | Yes | Yes | No | Yes | Yes | Yes | Yes | Yes | Yes | Yes | Good |
| Beadle, 2015 | Yes | Yes | No | No | Yes | Yes | Yes | Yes | Yes | Yes | Good |
| Best, 2021, Study2 | Yes | Yes | Yes | No | NA | NA | NA | NA | Yes | No | Fair |
| Bruine de Bruin, 2021 | Yes | Yes | Yes | Yes | Yes | NA | Yes | No | Yes | Yes | Good |
| Byrne, 2023, Study1 | Yes | Yes | Yes | Yes | NA | NA | NA | NA | Yes | No | Good |
| Byrne, 2023, Study2 | Yes | Yes | Yes | Yes | NA | NA | NA | NA | Yes | No | Good |
| Chapman, 2023 | Yes | Yes | No | No | NA | NA | NA | NA | Yes | No | Fair |
| Chi, 2023 | Yes | No | No | Yes | NA | NA | NA | NA | Yes | Yes | Fair |
| Cho, 2022 | Yes | Yes | Yes | Yes | No | Yes | Yes | Yes | No | Yes | Good |
| Cutler, 2021a | Yes | Yes | Yes | Yes | Yes | Yes | Yes | Yes | Yes | Yes | Good |
| Cutler, 2021b | Yes | Yes | Yes | Yes | NA | NA | NA | NA | Yes | Yes | Good |
| Dury, 2023 | Yes | Yes | Yes | No | NA | NA | NA | NA | Yes | No | Fair |
| Freund, 2014 | Yes | Yes | Yes | No | Yes | NA | Yes | No | Yes | Yes | Fair |
| Gaesser, 2017 | Yes | Yes | Yes | No | Yes | Yes | Yes | Yes | Yes | Yes | Good |
| Gingras, 2021 | Yes | Yes | Yes | No | Yes | Yes | Yes | Yes | Yes | Yes | Good |
| Gong, 2019 | Yes | Yes | Yes | Yes | NA | NA | NA | NA | Yes | Yes | Good |
| Gunderson, 2022 | Yes | Yes | Yes | Yes | Yes | NA | Yes | Yes | Yes | No | Good |
| Hao 2016 | Yes | Yes | No | No | Yes | Yes | Yes | Yes | Yes | Yes | Good |
| Jin, 2021 | Yes | Yes | Yes | No | NA | NA | NA | NA | Yes | No | Fair |
| Kettner, 2016 | Yes | Yes | No | No | NA | NA | NA | NA | Yes | Yes | Fair |
| Lakin, 1984 | Yes | Yes | No | No | NA | NA | NA | NA | Yes | No | Poor |
| Lockwood, 2021 | Yes | Yes | Yes | Yes | NA | NA | NA | NA | Yes | Yes | Good |
| Maxfield, 2014 | Yes | Yes | No | No | Yes | NA | Yes | NA | Yes | Yes | Fair |
| Midlarsky, 1989, Study1 | Yes | No | No | No | NA | NA | NA | NA | Yes | No | Poor |
| Midlarsky, 1989, Study2 | Yes | No | No | No | NA | NA | NA | NA | Yes | No | Poor |
| Mottus, 2015 | Yes | Yes | No | No | No | Yes | Yes | Yes | Yes | Yes | Fair |
| Nakavachara, 2018 | Yes | Yes | No | No | No | NA | NA | NA | No | Yes | Poor |
| Nava, 2023 | Yes | Yes | No | Yes | NA | NA | NA | NA | Yes | Yes | Good |
| Ogawa, 2020 | Yes | Yes | No | No | NA | NA | NA | NA | Yes | No | Fair |
| Ojha, 2014 | Yes | No | Yes | No | No | Yes | Yes | Yes | Yes | No | Fair |
| Pokorski, 2013 | Yes | Yes | No | No | No | Yes | Yes | Yes | Yes | No | Fair |
| Pollerhoff, 2022 | Yes | Yes | No | No | Yes | Yes | No | No | No | No | Fair |
| Pornpattananangkul, 2019 | Yes | Yes | Yes | Yes | Yes | Yes | Yes | Yes | Yes | No | Good |
| Rieger, 2015 | Yes | Yes | Yes | No | NA | NA | NA | NA | Yes | Yes | Good |
| Roalf, 2012 | Yes | Yes | Yes | No | Yes | Yes | Yes | Yes | Yes | Yes | Good |
| Roberts, 2019 | Yes | Yes | Yes | No | Yes | NA | Yes | NA | Yes | Yes | Good |
| Romano, 2021 | Yes | Yes | Yes | Yes | NA | NA | NA | NA | Yes | Yes | Good |
| Rosi, 2019 | Yes | Yes | Yes | No | Yes | Yes | Yes | Yes | Yes | Yes | Good |
| Ryan, 2022, Study 1 | Yes | Yes | Yes | Yes | NA | NA | NA | NA | Yes | No | Good |
| Ryan, 2022, Study 2 | Yes | Yes | Yes | Yes | NA | NA | NA | NA | Yes | No | Good |
| Ryan, 2022, Study 3 | Yes | Yes | Yes | Yes | NA | NA | NA | NA | Yes | No | Good |
| Ryan, 2023 | Yes | Yes | No | Yes | NA | NA | NA | NA | Yes | Yes | Good |
| Sawczak, 2019 | Yes | Yes | Yes | No | Yes | Yes | Yes | Yes | No | No | Fair |
| Sin, 2021 | Yes | Yes | Yes | No | No | Yes | Yes | Yes | Yes | Yes | Good |
| Sparrow, 2018, Study1 | Yes | Yes | Yes | No | Yes | Yes | Yes | Yes | Yes | Yes | Good |
| Sparrow, 2018, Study2 | Yes | Yes | Yes | No | Yes | Yes | Yes | Yes | Yes | Yes | Good |
| Sparrow, 2019 | Yes | Yes | No | Yes | Yes | Yes | Yes | Yes | Yes | Yes | Good |
| Sze, 2012 | Yes | Yes | Yes | No | Yes | Yes | Yes | Yes | Yes | Yes | Good |
| Zak, 2022 | Yes | Yes | No | Yes | Yes | NA | Yes | NA | Yes | Yes | Good |
